# Literary Notes

**Published:** 1910-03

**Authors:** 


### LITERARY NOTES

THE Cincinnati *Lancet-Clinic* appeared in new form on the first day of the New Year. Its columns, two to a page, now measure 3 x 9½, and its make-up is such as to introduce the reader to the editorial matter first. While we question the propriety of this latter change yet, on the whole, the magazine is improved very much in general appearance, and we trust it will reap an ample reward for its enterprising spirit.

W. B. SAUNDERS COMPANY, medical publishers of Philadelphia and London, have just issued a new edition—the thirteenth—of their handsome illustrated catalogue. It contains some twenty new books and new editions, and besides numerous black and white illustrations, there are two color cuts of special value. We strongly advise every physician to obtain a copy—sent for the asking. It will prove a ready guide to good medical books —books that all physicians need in their daily work.

